# Hospital Meetings, &c.

**Published:** 1898-03-12

**Authors:** 


### HOSPITAL MEETINGS, &C.

### THE NEW HOSPITAL FOR WOMEN.—ANNUAL MEETING.

The annual meeting of the governors and subscribers of the New Hospital for Women was held at the hospital on the 3rd inst. Sir Joshua Fitch took the chair. He made some introductory remarks pointing out that the support of hospitals was one of the most satisfactory forms of charity, and that the history of this particular institution illustrated as well the important advance made by women during the last quarter of a century. He advocated the cause of woman's higher education, as the cultivation of her intellect made her a more valuable citizen and a happier individual. He mentioned that he was one of the three trustees under the will of the late Mr. and Mrs. Pfeiffer, the whole of whose estate, amounting from £60,000 to £70,000, had been devoted to the institutions especially connected with the welfare of women. The New Hospital for Women benefitted to the extent of £3,000, which would be devoted to the erection of a nurses' home. The report, which was taken as read, was moved by Mr. Pollock, seconded by Mrs. Scharlieb, and carried unanimously. Mr. Scharlieb said that the hospital had not been quite so fortunate financially this year as usual. They had always endeavoured to be honest and pay their way, but there was a small deficit, and unless more help was obtained there must be a larger one next year. The work could not go backward. Every one of their forty beds had been full, and there were as a rule fifty patients waiting their turn to come in. The sad part of it was that some were suffering from diseases which necessitated almost immediate operation, for if once the favourable moment passed little could be done for them. To admit such patients meant beds, and beds meant money. They would fain be without anxiety on this count, and she trusted that their friends would do their best and beg hard for them. Some hospitals employed an admirable officer known as an almoner, whose duty it was to inquire into the affairs of the patients. She thought that the institution of a professional beggar for their hospital desirable as they had degenerated, particularly since the days of Mrs. Anderson, who had a wonderful way of coaxing the guineas and more than that, the cheques for large amounts, out of the pockets of the wealthy. Mrs. Garrett Anderson said a few words as to the aim of the hospital being to do work of the highest degree of excellence rather than a large quantity indifferently, and pointed out that success was always the result of care and constant looking after things, and of not being too content with things as they were. Mrs. Boyd drew attention to the fact that the hospital was not a hospital for the special diseases of women but a hospital for the general disease in women. The late chief secretary of the Ameer of Afghanistan, Ch. S. Muhammed Khan, drew a parallel between the East and the West and the manner in which the women regarded women doctors. In the East medical women were obtaining an increasing reputation both as regards their treatment of women and men. Miss Hamilton, M.D., medical adviser to the Ameer, had cured his Highness of a disease that had baffled his native physicians, and was now doing excellent work in establishing hospitals and vaccination stations. Mary, Lady Hobart, was elected vice-president, and Mr. F. J. Dryhurst was elected to the Managing Committee. The usual votes of thanks were carried. During the year 555 patients have been treated; 17 made use of the two private wards. There have been 77 major operations; 6,831 letters have been issued to out-patients, and nearly all have been renewed. The maternity department has attained most successful results. The receipts this year, including a legacy of £1,000, are £5,013; the expenditure has been £4,965, and there is still a debt of £100, to be paid which is owing to the building fund. In addition to the nurses' home, it is proposed to remodel the present quarters of the nurses for the purpose of opening five cancer beds which have been endowed by the Rev. James Chadburn to the extent of £5,000. Another friend has offered £500 to build an isolation ward. A large operating theatre is also to be built and a room will be set apart for the administration of anæsthetics.

### NATIONAL HOSPITAL FOR DISEASES OF THE HEART AND PARALYSIS.

The Earl of Verulam presided at the forty-first annual meeting of the National Hospital for Diseases of the Heart and Paralysis, Soho Square, on Thursday, 3rd inst. The report presented gave the income of the general fund from all sources for 1897 as £2,006, and the expenditure as £2,134, comparing with £1,969 and £2,163 respectively in 1896. In addition to this £1,000 had been received in legacies, the major part of which had been invested. Many subscriptions and donations had been diverted to the numerous Jubilee funds, but the deficiency had been met by a gift of £300 from Mrs. Eliza Turner and the increase of contributions from other subscribers, so that the hospital had been maintained in full working order. The amounts received from the Hospital Saturday and Sunday Funds were respectively £64 5s. and £52 10s. From the Prince of Wales' Fund £52 10s. had been received. The committee expressed the hope that the suggestion that grants from the last-named fund should in future be given only to institutions having at least 100 beds would not be carried out. During the year the attendances of out-patients numbered 10,079. There were 141 admissions as in-patients. These figures compared with 9,767 and 144 in 1896. In moving the adoption of the report, the Chairman alluded to the Prince of Wales's Fund, which had raised a handsome sum, but not enough to carry out to the full the wishes of his Royal Highness. He hoped the rule as to the number of beds required before a hospital could participate would not be rigidly adhered to, otherwise some useful institutions would suffer. Canon Floyd referred to the comparatively small number of in-patients received, and said this was due to the fact that their special desire was not to make a good showing, but to treat their patients well, and give full time and care to ensure complete recovery.

### ROYAL WESTMINSTER OPHTHALMIC HOSPITAL.

Sir Charles A. Turner presided over the annual general meeting, held at the hospital on Friday, March 4th. According to the annual report the number of in-patients treated in 1897 was 644, compared with 558 in the previous year. The new out-patients numbered 10,588, and their total attendances registered 26,404. For some time past the committee have been considering the question of increased accommodation for carrying on the work of the hospital, and the response to their appeal for funds being inadequate, it has been decided to hold a dinner in aid of the building fund on April 30th next at the Whitehall Rooms, when the Speaker of the House of Commons will preside. The past year has been a satisfactory one from a financial point of view. The income of the general fund amounted to £2,661, as against £2,251 in 1896, and the expenditure to £2,262, as compared with £2,386. In addition, legacies came to hand to the amount of £3,276, of which £1,586 had been invested and assigned to the sustentation fund, and £1,635 to the building fund. The Chairman, after congratulating the governors on the support rendered to the institution in the jubilee year, commented on the fact that during the last forty or fifty years London had seen the founding of several institutions similar to their own. The growth of the hospital, however, had been remarkable. In 1850 the total number of patients treated at the institution was 4,954, of whom 166 were in-patients. Last year, as the report showed, there were 644 in-patients and 10,588 out-patients, and the need for an ophthalmic hospital was far greater now than in 1850. The experience of Mr. Brudenell Carter with regard to the eyes of city-bred children had been confirmed by the experience of the medical officers of this institution. The effect of study on badly-nourished children was to considerably increase the number of the young who were compelled to seek relief at the hospital. Sir Charles proceeded to remark that, although in point of economy the hospital would compare favourably with the best-managed institutions, they had been unable to impress the Hospital Sunday Fund with the importance of recognising economical administration as the first claim to help. They found themselves in the position of receiving less than institutions which gave no more to the public than they did, especially in ophthalmic cases. When he pointed out to the Sunday Fund Committee the small amount of the domestic expenses, it was suggested that they starved their patients. He thereupon invited the members of the committee to call in any day to see for themselves. But, of course, it was obvious that if there were any ground for complaint the medical staff would at once bring the matter to the notice of the committee, and he might say, as a fact, that no patient had ever complained on this score. In seconding the adoption of the report, Mr. Henry Power (deputy-chairman), remarking on the increase in the number of patients, said that many years ago there might have been some hocus-pocus in preparing the figures, but to-day the statistics were absolutely to be relied upon. The formal resolutions were unanimously adopted.

### ST. PETER'S HOSPITAL.

The annual meeting of St. Peter's Hospital for stone was held at the hospital on Thursday, March 3rd, Mr. F. A. Bevan presiding. During 1897 430 in-patients were admitted, and of this number 389 were cured or relieved, 30 discharged unrelieved, while 11 died, several of the latter being of advanced years. A table prepared by the house-surgeon showed the death-rate under operations for stone in each decade since the opening of the hospital. For the period 1864-73 the death-rate was 15·5 per cent.; for 1874-83, 15 30 per cent.; for 1884-93, 8·29 per cent.; while for the four years, 1894-97, it was only 3·17 per cent. The decrease in the mortality in recent years was mainly attributed to the substitution of litholapaxy for the older methods of operating. The attendance of out-patients increased during 1897, there having been 4,858 new patients, as against 4,799 and 32,771 attendances as compared with 32,717. The receipts for the year amounted to £3,237, including £2,272 from patients, and the expenditure to £3,567. The Chairman, in dealing with the deficit, said that the hospital, in common with other hospitals, had suffered through the diversion of contributions to the Prince of Wales's Hospital Fund, and the consequence was that they would be compelled to realise some of their investments unless the subscriptions and donations increased this year. Referring to the £2,066 contributed by out-patients, derived from over 32,000 attendances, Mr. Bevan observed that when it was suggested to those seeking relief that they might contribute towards the out-of-pocket expenses the patients cheerfully responded, and in his opinion they constituted a deserving class who ought to be relieved. Some remarks had been made recently upon the need of very close scrutiny of the hospitals. They were not afraid of any scrutiny, said the chairman; indeed, the more closely their affairs were looked into the more would the hospital be appreciated. Mr. Hurry Fenwick, F.R.C.S. (honorary surgeon), called attention to the fact that the hospital was a school for rising surgeons, who could attend operations and obtain any information they required. The fact that 440 medical practitioners and students attended during the past year was a further illustration of the value attached to the work. After the adoption of the report it was resolved that for the regulation requiring the surgeons to be Fellows of the Royal College of Surgeons of England the following be substituted: "The surgeons must be Fellows of one of the Royal colleges or masters in surgery of a University in the United Kingdom, duly registered, and not practising pharmacy or midwifery either alone or in partnership."

### THE HOME FOR CONFIRMED INVALIDS.

At 36, Aubert Park, Highbury, there is a home for poor ladies who are confirmed invalids. Each possesses a little money, and pays from 12s. 6d. to 15s. weekly for the really homelike home provided. After inspecting the conveniently arranged, roomy house, noting all the invalid requirements provided, and chatting with the capable and pleasant nurse, who, with three probationers and a matron, take such excellent care of the invalids, it is a pleasant surprise to find that the Home once built is very nearly self-supporting—a fact that speaks well for the economy in the management. There is another fact speaking equally well for the care bestowed on the patients, namely, that one lady was an inmate for fifty years, and another, who died lately, had been there twenty-four years, whilst there are two others who have resided in the home for longer still.

At a drawing-room meeting on the 8th inst., at which the Bishop of Stepney and many local friends of the Home were present, an earnest appeal was made to raise the sum of 75,000 to adapt and equip a house in Highbury Terrace, of which the freehold has been acquired. About 150 eligible candidates are waiting vacancies—that is, candidates are waiting five deep for every bed. Dr. Stokes, the hon. medical attendant, remarked in the course of the proceedings that these were cases from which the medical practitioner did not reap much glory. Before the patients came to the Home they had tried many remedies and done everything that could be done, and all that remained was to watch them carefully and relieve actual pain. Though doctors could do little, good nursing and suitable, well-cooked food at regular hours could do much, apart from the advantages of society and co-operation afforded by the Home. The Bishop of Stepney put forth two pleas for the Home. The first, because it was strongly supported locally, which was always a guarantee that the work was a good one; secondly, because it was an example of the inventive power of Christianity. The gathering together of these poor ladies, who lived in the shadow of death, and making their lives comfortable and cheerful, was a true charity, and charity was twice blessed, the givers sharing equally in the benefits with the recipients.

After a successful meeting a collection was made, which resulted in £30 being added to the £1,467 already gathered towards the desired £5,000.

